# A critical role of a plant-specific TFIIB-related protein, BRP1, in salicylic acid-mediated immune response

**DOI:** 10.3389/fpls.2024.1427916

**Published:** 2024-07-30

**Authors:** Binjie Xu, Baofang Fan, Zhixiang Chen

**Affiliations:** ^1^ Department of Botany and Plant Pathology, Purdue University, West Lafayette, IN, United States; ^2^ Key Laboratory of Southwestern Chinese Medicine Resources and Innovative Institute of Chinese Medicine and Pharmacy, Chengdu University of Traditional Chinese Medicine, Chengdu, Sichuan, China

**Keywords:** TFIIB-related proteins, salicylic acid, plant immunity, isochorismate synthase 1, transcription factors, pathogenesis-related (PR) genes

## Abstract

An integral part of plant immunity is transcription reprogramming by concerted action of specific transcription factors that activate or repress genes through recruitment or release of RNA polymerase II (Pol II). Pol II is assembled into Pol II holoenzyme at the promoters through association with a group of general transcription factors including transcription factor IIB (TFIIB) to activate transcription. Unlike other eukaryotic organisms, plants have a large family of TFIIB-related proteins with 15 members in Arabidopsis including several plant-specific TFIIB-related proteins (BRPs). Molecular genetic analysis has revealed important roles of some BRPs in plant reproductive processes. In this study, we report that Arabidopsis knockout mutants for BRP1, the founding member of the BRP protein family, were normal in growth and development, but were hypersusceptible to the bacterial pathogen *Psuedomonas syringae*. The enhanced susceptibility of the *brp1* mutants was associated with reduced expression of salicylic acid (SA) biosynthetic gene *ISOCHORISMATE SYNTHASE 1* (*ICS1*) and SA-responsive *PATHOGENESIS-RELATED* (*PR*) genes. Pathogen-induced SA accumulation was reduced in the *brp1* mutants and exogenous SA rescued the *brp1* mutants for resistance to the bacterial pathogen. In uninfected plants, BRP1 was primarily associated with the plastids but pathogen infection induced its accumulation in the nucleus. BRP1 acted as a transcription activator in plant cells and binded to the promoter of *ICS1*. These results collectively indicate that BRP1 is a functionally specialized transcription factor that increasingly accumulates in the nucleus in response to pathogen infection to promote defense gene expression.

## Introduction

In most eukaryotes, three multi-subunit RNA polymerases (Pol I, II and III) are responsible for the transcription of nuclear genome (Vannini and Cramer, 2012). Pol II, which transcribes genes encoding mRNAs, small nuclear RNAs (snRNAs) and microRNA (miRNAs), has been most extensively studied for understanding transcription and transcriptional regulation. Pol II requires up to seven different general transcription factors (TATA box-binding protein or TBP, transcription factor IIA or TFIIA, TFIIB, TFIID, TFIIE, TFIIF and TFIIH) for transcription initiation (Cox et al., 2012; Vannini and Cramer, 2012). These general transcription factors recognize promoter elements, recruit and assist Pol II in DNA opening and initial RNA synthesis (Archuleta et al., 2024). Pol I and III, which synthesize 25S ribosomal RNA (rRNA) and small untranslated RNAs (tRNA and 5S rRNA), respectively, also require the same or similar general transcription factors for transcription initiation (Vannini and Cramer, 2012). Thus, each of these general transcription factors often has 2 to 4 paralogs. In yeast (*Saccharomyces cerevisiae*), TFIIB, a Pol II general transcription factor, has two paralogs, Rrn7 and Brf-1, as general transcription factors for Pol I and III, respectively (Knutson, 2013). On the other hand, there are thousands of gene-specific transcription factors in a typical eukaryotic organism that control complex tissue- and cell-specific gene expression. Unlike general transcription factors, many gene-specific transcription factors belong to large families of many members with both shared and distinct functions (Shiu et al., 2005; Qu and Zhu, 2006; Charoensawan et al., 2010; Moore and Goldberg, 2011; Catarino et al., 2016).

In addition to the three conserved Pols, plants contain Pol IV and V, which are required for small interfering RNAs (siRNA) biogenesis, siRNA targeting and RNA-directed DNA methylation (Ream et al., 2009; Huang et al., 2015). Plants also contain multiple copies of TBP with two in Arabidopsis (Heard et al., 1993). The largest number of plant general transcription factors belong to the TFIIB-related protein (BRP) family with 15 in Arabidopsis (Knutson, 2013; Ning et al., 2021). Phylogenetic analysis indicates that the expanded TFIIB-related protein family in plants can be grouped into five distinct subfamilies, three of which correspond to the TFIIB, Rrn7, and Brf clades conserved in all eukaryotes (Knutson, 2013). There are two additional TFIIB-related protein subfamilies, named BRP1 and BRP5, in plants that are not present in other eukaryotes (Knutson, 2013). Genetic studies have shown that mutants for Arabidopsis TFIIB1 (Zhou et al., 2013b), TFIIB2 (Zhou et al., 2013b), BRP2 (Cavel et al., 2011), BRP4 (Qin et al., 2014), BRP5 (Niu et al., 2013) and Maternal Effect Embryo Arrest 12 (MEE12) (Chen et al., 2007) are defective in pollen and/or endosperm development, indicating that they are regulators of plant reproductive processes. In addition, Arabidopsis MEE65 is a Rrn7 homolog for Pol I (Burton and Burton, 2014), while Arabidopsis MEE12 is a close Brf homolog for Pol III. The embryo arrest phenotype of *mee12* and *mee65* could be due to defective Pol I and III transcription, respectively.

Arabidopsis BRP1 was first described more than 20 years ago as a plant-specific TFIIB-related protein (originally named pBRP) based on its conserved TFIIB structural features and the ability to bind TBP (Lagrange et al., 2003). Intriguingly, Arabidopsis BRP1 was primarily localized to the cytoplasmic surface of the plastid envelope and accumulated in the nucleus only after chemical inhibition of the proteasome activity or in the *fusca 6* (*fus6*) mutant deficient in the CONSTITUTIVE PHOTOMORPHOGENESIS 9 (COP9) signalosome, which targets proteasome-mediated degradation of transcription factors (Lagrange et al., 2003). Thus, Arabidopsis BRP1 is subject to rapid turnover in the nucleus by proteasome-mediated protein degradation. It has been proposed that plant BRP1 is a general transcription factor for Pol I but not for Pol II or III based on the types of promoters recognized by BRP1 from red algae *Cyanidioschyzon merolae* and Arabidopsis (Imamura et al., 2008). Chromatin immunoprecipitation (ChIP) analysis revealed that CmpBRP1 specifically recognized the rDNA promoter region *in vivo*, and the occupancy was correlated to *de novo* 18S rRNA synthesis (Imamura et al., 2008). On the other hand, BRP1 did not recognize the Pol II-dependent promoters of five light-responsive protein-coding genes or the Pol III-dependent 5S rDNA promoter (Imamura et al., 2008). Pol I-dependent transcription from the rDNA promoter in crude cell lysate was inhibited by the CmpBRP1 antibody or when the CmpBRP1–CmTBP binding site in the rDNA promoter was mutated (Imamura et al., 2008). It was also shown that CmpBRP1 co-immunoprecipitated and co-localized with the Pol I subunit, CmRPA190, in the cell (Imamura et al., 2008).

Other studies, however, have provided strong evidence for a role of Arabidopsis BRP1 in the transcription of protein-coding genes by Pol II. Arabidopsis BRP1 interacted with *Agrobacterium* transcription activator Virulence E3 (VirE3) and had a strong effect on VirE3-activated expression of protein-coding genes in plants (Garcia-Rodriguez et al., 2006; Niu et al., 2015; Li et al., 2021a). One of the strongly activated genes by VirE3 encodes VirE2-interacting Protein 1(VIP1)-binding F-box Protein (VBF; At1G56250), which affected the levels of VirE2 and VIP1 (Niu et al., 2015; Li et al., 2021a). In Arabidopsis cells, co-expression of VirE3 induced accumulation of BRP1 in the nucleus and co-expression of BRP1 enhanced VirE3-stimulated transcription of *VBF* (Niu et al., 2015). These results indicate that VirE3 targets the transcriptional machinery of plant cells to promote plant transformation by *Agrobacterium* (Niu et al., 2015). More importantly, BRP1 promoted VirE3-mediated transcription of a large number of protein-coding genes in Arabidopsis (Niu et al., 2015; Li et al., 2021a). These findings strongly indicate that plant-specific BRP1 functions in the transcription of protein-coding genes by Pol II.

We have been studying the roles of important protein quality control pathways including autophagy in plant responses to both biotic and abiotic stresses. We became interested in BRP1 because proteomic profiling revealed that it was elevated in the double mutants for the selective autophagy receptor Neighbor of BRCA1 (Breast Cancer Gene 1) Gene 1 (NBR1) and the chaperone-dependent ubiquitin E3 ligase Carboxy-terminal Heat Shock Protein 70-interacting Protein (CHIP) (Zhou et al., 2013a, 2014). To analyze the biological functions of BRP1, we generated *brp1* knockout mutants and found them to be normal in growth and development. However, these *brp1* mutants were hypersusceptible to the bacterial pathogen *Psuedomonas syringae*. Thus, unlike other characterized TFIIB-related proteins with critical roles in plant growth and development, BRP1 has an important role in plant immunity. To understand the molecular basis for the critical role of BRP1 in plant immunity, we compared wild-type (WT) and *brp1* mutant plants for pathogen-induced expression of salicylic acid (SA) biosynthetic gene *ISOCHORISMATE SYNTHASE 1* (*ICS1*) and SA-responsive *PATHOGENESIS-RELATED* (*PR*) genes. We also analyzed pathogen-induced accumulation of SA and the effects of exogenous SA on the disease resistance of the *brp1* mutants. We further investigated pathogen-induced nuclear accumulation and the transcription regulatory activity of BRP1, as well as the direct binding to defense-related gene promoters by BRP1 in plant cells. These results collectively indicate that BRP1 plays a critical role in plant immunity through increased nuclear accumulation upon pathogen infection to promote gene expression associated with SA-mediated defense responses.

## Materials and methods

### Plant materials and growth conditions

Arabidopsis (*Arabidopsis thaliana*) WT and mutant plants used in the study are all in the *Col-0* background. Homozygous T-DNA insertion mutants *brp1-1* (WiscDsLoxHs064_04H) and *sa induction deficient2* (*sid2;* Salk_133146) were identified by PCR using primers flanking the T-DNA insertions listed in **Supplementary Table 1**. Arabidopsis were grown in growth chambers or rooms at 24°C, 120 µmol m^-2^s^-1^ light on a photoperiod of 12-hour light and 12-hour dark.

### Generation of *brp1-2* mutant using CRISPR/Cas9 genome editing

A site in the second exon of the *BRP1* gene, which is about 200 nucleotides from the 5’-end of its coding sequence, was selected as a target for genome editing. The target sequences (ATTGAAGGCGGTAATGAATCCGGT and AAACACCGGATTCATTACCGCCTT) were inserted into a plant CRISPR/Cas9 vector containing the *Cas9* gene driven by the promoter of the *YAO* gene, which is preferentially expressed in the tissues undergoing cell division (Yan et al., 2015). Arabidopsis transformation was performed using the floral dipping method (Clough and Bent, 1998). For identification of mutations in the target site, the region was PCR-amplified using PCR primers flanking the target site (**Supplementary Table 1**) at T2 generation and directly sequenced.

### Disease resistance assays

Assays of Arabidopsis plant resistance to a virulent strain of *Pseudomonas syringae* pv *tomato* DC3000 (*Pst*DC3000) were performed as previously described (Wang et al., 2014, 2015).

### Real-time quantitative PCR

Total RNA was isolated from WT and mutant leaves using Trizol reagent (Invitrogen) and treated by DNase with Turbo DNA-free (Thermo Fisher Scientific) to remove contaminated DNA. cDNA was synthesized from 2.5 μg total RNA using SuperScript III reverse transcriptase (Invitrogen). Transcript levels were determined by RT-qPCR with *ACTIN2* as an internal control using gene-specific primers (**Supplementary Table 2**) as previously described (Li et al., 2021b).

### Assays of SA levels

Free and total SA content was determined with a biosensor strain *Acinetobacter* species, ADPWH_lux, as described previously (Defraia et al., 2008). SA concentrations in the leaf samples were calculated based on the SA standard curve, which was constructed using the *sid2* mutant leaf extract (Defraia et al., 2008).

### Epitope-tagged BRP1 fusion construct and transgenic plants

The genomic sequence of *BRP1* including its ~2.0 kb promoter was PCR-amplified using gene-specific primers (agcggcgcgccAGCGTTTGGGGTTTCTCACT and agcttaattaaGAAGTCTCCATGGGGATTATCAG). The amplified *BRP1* genomic sequence was fused with a 4x myc epitope tag in a plant transformation vector as previously described (Wang et al., 2019). The construct was introduced into Arabidopsis plants using floral dipping method (Clough and Bent, 1998).

### Chloroplast and nuclear isolation

Chloroplasts were isolated from leaves of transgenic plants expressing myc-tagged *BRP1* using a chloroplast isolation kit (Sigma-Aldrich, USA). Procedures for homogenization, removal of cell debris and leaf tissue by filtration, collection of total cell chloroplast fraction by centrifugation, and separation of intact chloroplasts using Percoll gradient were performed following the manufacturer’s protocol.

Nuclei were isolated from Arabidopsis leaves using a plant nuclei isolation kit CelLytic™ PN (Sigma-Aldrich, USA). Nuclei were first prepared from leaves with the nuclei isolation buffer provided in the kit. After mesh filtering, the cell lysate was centrifuged with 2.3 M sucrose at 12,000xg for 10 min and the nuclei pellet was collected by following the manufacturer’s protocol.

### Protein extraction and blotting

Total proteins from Arabidopsis leaves, isolated chloroplast and nuclei were isolated in a protein extraction buffer (50 mM Tris-HCl, pH 7.5, 150 mM NaCl, 5 mM EDTA, 0.1% Triton X-100, 0.2% Nonidet P-40, 0.6 mM phenylmethylsulfonyl fluoride, 80 μM MG115, 80 μM MG132) and complete protease inhibitor cocktail tablet (Roche, USA). Protein isolation, electrophoretic separation, blotting and detection of BRP1-myc proteins using anti-myc antibodies were performed as previously described (Li et al., 2021b). Proteins isolated from chloroplast and unclei were also analyzed by protein blotting using antibodies against chloroplast-specific PsbH and nucleus-specific histone H4 proteins to assess the extent of cross-contamination. Anti-PsbH and histone H4 antibobies were obtained from Agrisera and Abcam, respectively.

### Assays of transcriptional regulatory activity of BRP1

Transgenic *Arabidopsis* plants containing a β-glucuronidase (*GUS*) reporter gene driven by a synthetic promoter consisting of the −100 minimal *Cauliflower Mosaic Virus* (*CaMV*) *35S* promoter and eight copies of the *LexA* operator sequence have been described previously (Kim et al., 2006). To generate effector genes, the DNA fragment for the LexA DBD was digested from the plasmid pEG202 (Clontech) using *Hin*dIII and *Eco*RI and cloned into the same sites in pBluescript. The full-length *BRP1* coding sequence was subsequently subcloned behind the *LexA DBD* to generate a translational fusion. The *LexA DBD-BRP1* fusion genes were cloned into the *Xho*I and *Spe*I sites of pTA2002 behind the steroid-inducible promoter (Aoyama and Chua, 1997). As controls, the unfused *LexA DBD* and *BRP1* genes were also cloned into the same sites of pTA7002. These effector constructs were directly transformed into the transgenic *GUS* reporter plants, and double transformants were identified through screening for antibiotic (hygromycin) resistance. Determination of the activation or repression of *GUS* reporter gene expression by the effector proteins was performed as described previously (Kim et al., 2006).

### ChIP-qPCR

Six-week-old transgenic plants expressing myc-tagged BRP1 under its native promoter were inoculated with *Pst*DC3000. Leaf samples were collected at 0 and 24 hours post inoculation (hpi) and processed as previously described (Birkenbihl et al., 2012). After cross-linking by vacuum infiltration of 1% formaldehyde solution, nuclei were isolated and sonicated. The sheared chromatin was incubated with anti-myc antibodies (ChIP grade; ABCAM). Immuncomplexes were collected with protein A-agarose and DNA was extracted and precipitated after reversing crosslinking. qPCR was performed using gene-specific primers (**Supplementary Table 3**).

## Results

### Hyper-susceptibility of *pbrp1 mutants* to *P. syringae*

**Figure 1:**
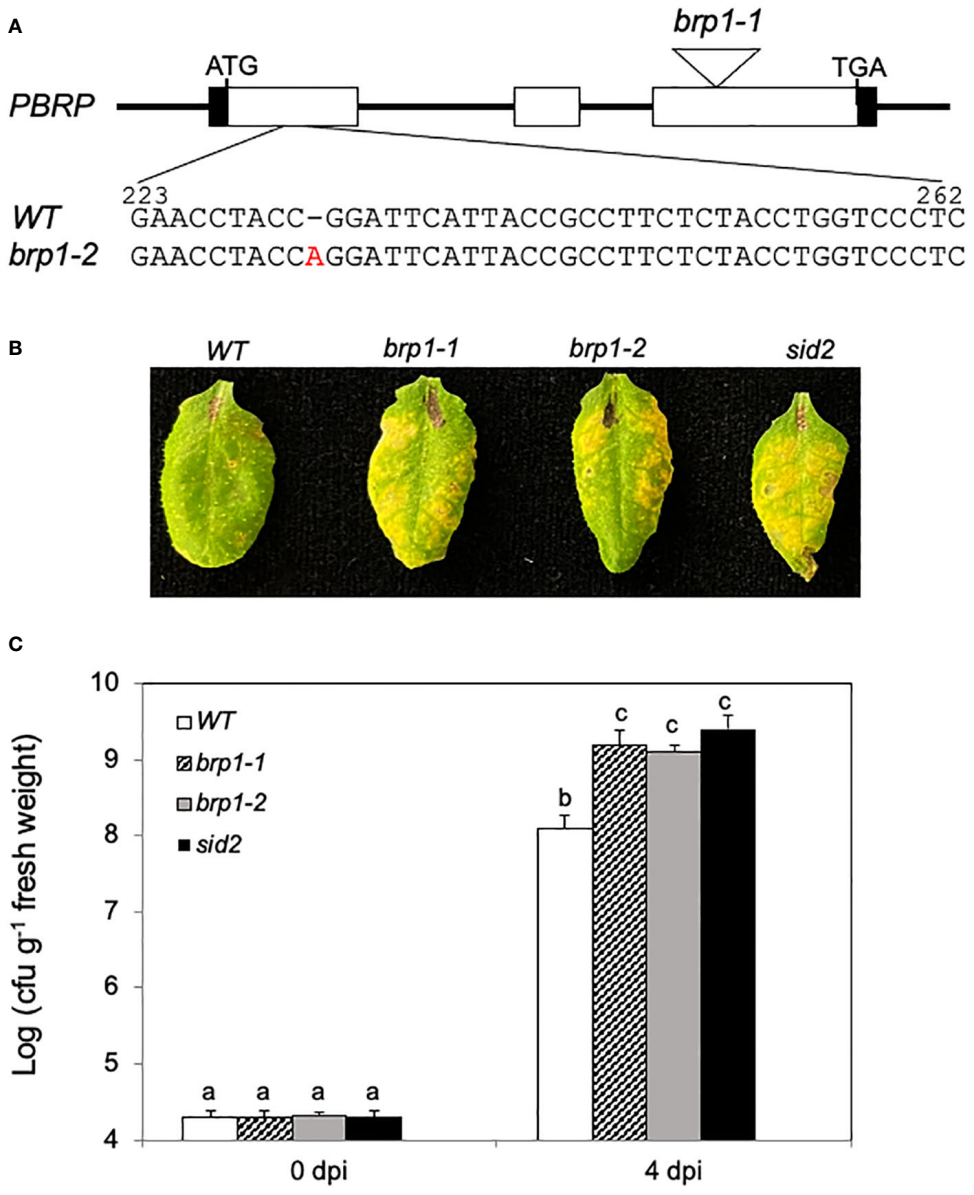
Compromised disease resistance of *brp1* mutants to *Pst*DC3000. **(A)** Arabidopsis *BRP1* gene structure and mutants. The *brp1-1* T-DNA mutant contains a T-DNA insertion in the third exon of *BRP1*. The *brp1-2* mutant contains a single A base insertion between nucleotides 232 and 233 of the *BRP1* coding sequence. **(B)** Disease symptom development after infection by the virulent *Pst*DC3000. Leaves of 6 weeks old Arabidopsis Col-0 WT, *brp1* and *sid2* mutant plants were infiltrated with *Pst*DC3000 (OD_600_ = 0.0002 in 10 mM MgCl_2_). Pictures of representative leaves were taken at 4 dpi. **(C)** Leaf samples were taken at 0 or 4 dpi to determine the bacterial growth. The means and standard errors were calculated from 10 plants (n=10) for each genotype. According to Duncan’s multiple range test (P=0.01), means of the values do not differ if they are indicated with the same letter. The experiment was repeated three times with similar results.

To determine the role of Arabidopsis BRP1 directly, we isolated a *brp1* T-DNA mutant (*brp1-1*) that contains a T-DNA insertion in the third exon (**Figure 1A**). RT-qPCR showed that the *pbrp-1* mutant had little detectable full-length *BRP1* transcripts (**Supplementary Figure 1A**). We also generated a second *brp* mutant (*brp1-2*) by targeting a site at the N-terminal domain of the BRP protein using CRIPR/cas9-mediated genome editing. The *brp1-2* mutant contains a single A base insertion between nucleotides 232 and 233 of the *BRP1* coding sequence (**Figure 1A**). This single nucleotide insertion in the *brp1-2* mutant causes a reading frame shift and introduces a premature termination codon that is expected to produce a protein of 106 amino acid residues (**Supplementary Figure 1B**). Both *brp1-1* and *brp1-2* mutants grew and developed normally. In addition, the levels of rRNAs in the *brp1* mutants based on the stained band intensities after electrophoretic separation of total RNA were not significantly reduced as compared to those in WT (data not shown).

To analyze the response of the *brp1* mutants to pathogen infection, we compared them with Col-0 WT and a SA-deficient *sid2* mutant for response to the virulent bacterial pathogen *Pst*DC3000. As shown in **Figure 1B**, at 4 days post inoculation (dpi), WT plants developed very mild symptoms of chlorosis. On the other hand, the two *brp1* mutants developed more severe disease symptoms at 4 dpi than WT plants (**Figure 1B**). The enhanced disease symptoms in the *brp1* mutants after *Pst*DC3000 infection were similar to those in the *sid2* mutant (**Figure 1B**), which accumulated greatly reduced levels of SA (Wildermuth et al., 2001; Garcion et al., 2008). The levels of bacterial growth in the *brp1* and *sid2* mutants were also 10 to 20 times higher than those in the WT plants (**Figure 1C**). Thus, the *brp1* mutants were as susceptible to the bacterial pathogen as SA-deficient *sid2* mutant plants (**Figure 1**).

### Defects in pathogen-induced defense genes in *brp1* mutants

**Figure 2:**
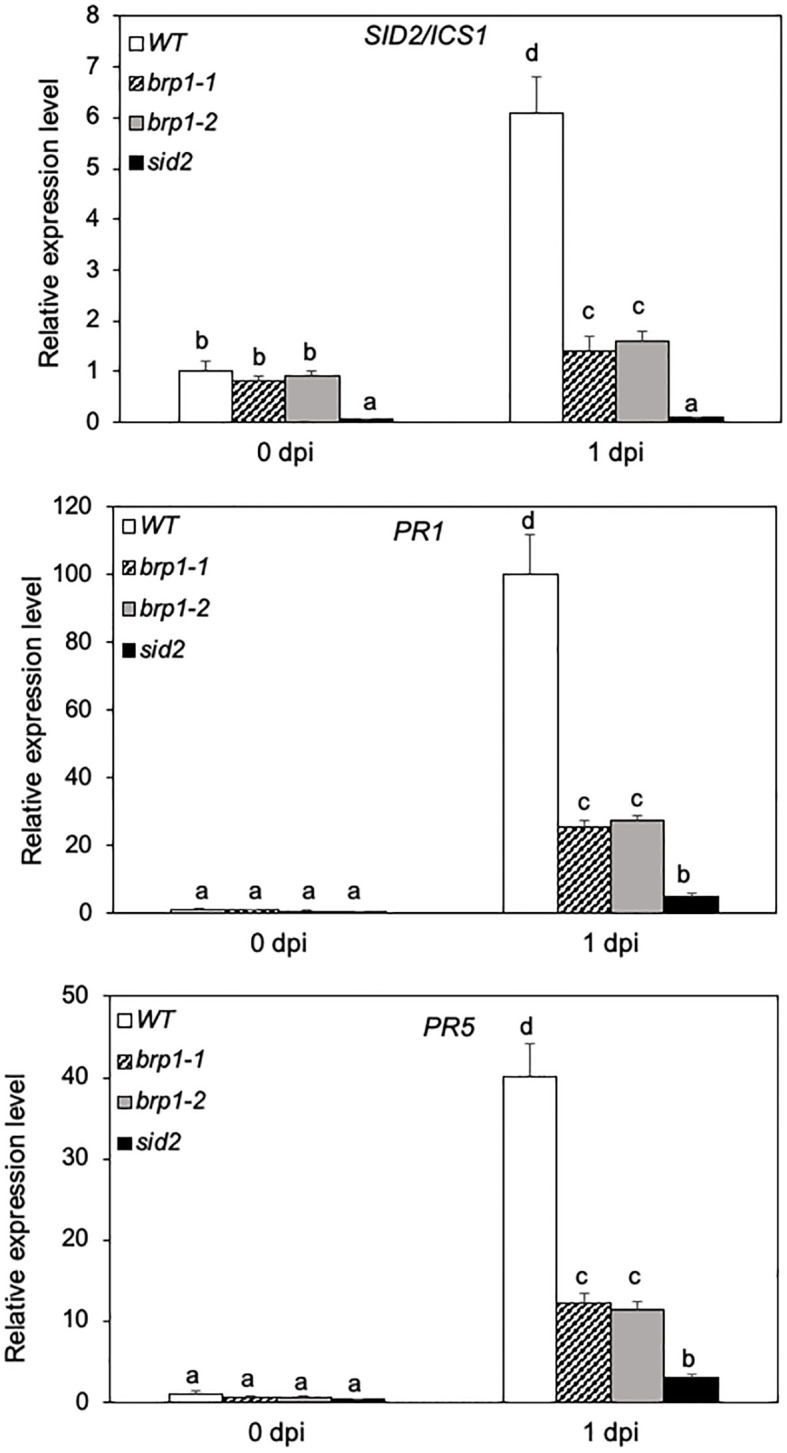
Reduced defense gene expression in pathogen-infected *brp1* mutants. Leaves of 6 weeks-old Arabidopsis Col-0 WT, *brp1* and *sid2* mutant plants were infiltrated with *Pst*DC3000 (OD_600_ = 0.0002 in 10 mM MgCl_2_). Total RNA was isolated from leaf samples collected at indicated dpi. Transcript levels of *ICS1/SID2*, *PR1* and *PR5* were determined using RT-qPCR. Error bars indicate SE (n = 3). According to Duncan’s multiple range test (P=0.01), means of the values do not differ if they are indicated with the same letter. The experiment was repeated twice with similar results.

SA-mediated defense signaling is important for resistance to *P. syringae* in Arabidopsis (Glazebrook, 2005). To analyze the molecular basis for enhanced susceptibility of the *brp1* mutants to the bacterial pathogen, we compared WT, *brp1* and *sid2* mutants for the expression of *SID2*, which codes for SA biosynthetic enzyme ICS1 (Wildermuth et al., 2001), before and after *Pst*DC3000 infection. At 0 dpi, no significant difference was observed in the transcript levels of *SID2* between WT and the two *brp1* mutants (**Figure 2**). After inoculation with *Pst*DC3000, the levels of *SID2* transcripts were increased by more than 6-fold in WT but only about 1.5-fold in the *brp1* mutants by 1 dpi (**Figure 2**). Thus, pathogen-induced *SID2* expression was compromised in the *brp1* mutants. As expected, little expression of *SID2* was detected either prior to or after *Pst*DC3000 infection in the *sid2* mutant due to disruption of the analyzed gene by a T-DNA insertion (**Figure 2**).

We also compared the expression of SA-regulated *PR* genes in WT, *brp1* and *sid2* mutants. In WT, *PR1* and *PR5* transcripts were elevated by more than 100- and 40-fold, respectively, during the first dpi (**Figure 2**). In the *brp1* mutants, however, there was only about 25- and 10-fold increase in the *PR1* and *PR5* transcripts, respectively, during the first dpi (**Figure 2**). Thus, pathogen-induced *PR* gene expression was also compromised in the *brp1* mutants. The levels of transcripts for pathogen-induced *PR1* and *PR5* genes were even further reduced in the *sid2* mutant when compared to those in WT and *brp1* mutants (**Figure 2**).

### Reduction in pathogen-induced SA accumulation in *brp1* mutants

**Figure 3:**
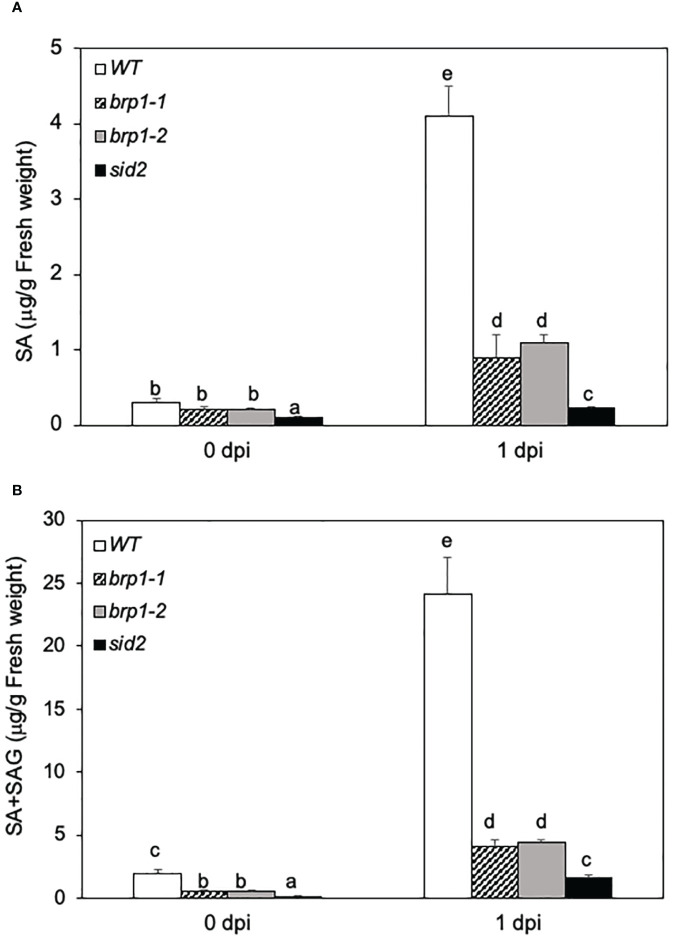
Reduced SA levels in the *brp1* mutants.Leaves of 6 weeks-old Arabidopsis Col-0 WT, *brp1* and *sid2* mutant plants were infiltrated with PstDC3000 (OD^600^ = 0.0002 in 10 mM MgCl^2^). Inoculated leaves were sampled at indicated dpi and determined for both free SA **(A)** and free SA plus SAG **(B)** content using a bacterial SA biosensor. Error bars indicate SE (n = 5). According to Duncan’s multiple range test (P=0.01), means of the values do not differ if they are indicated with the same letter.

**Figure 4:**
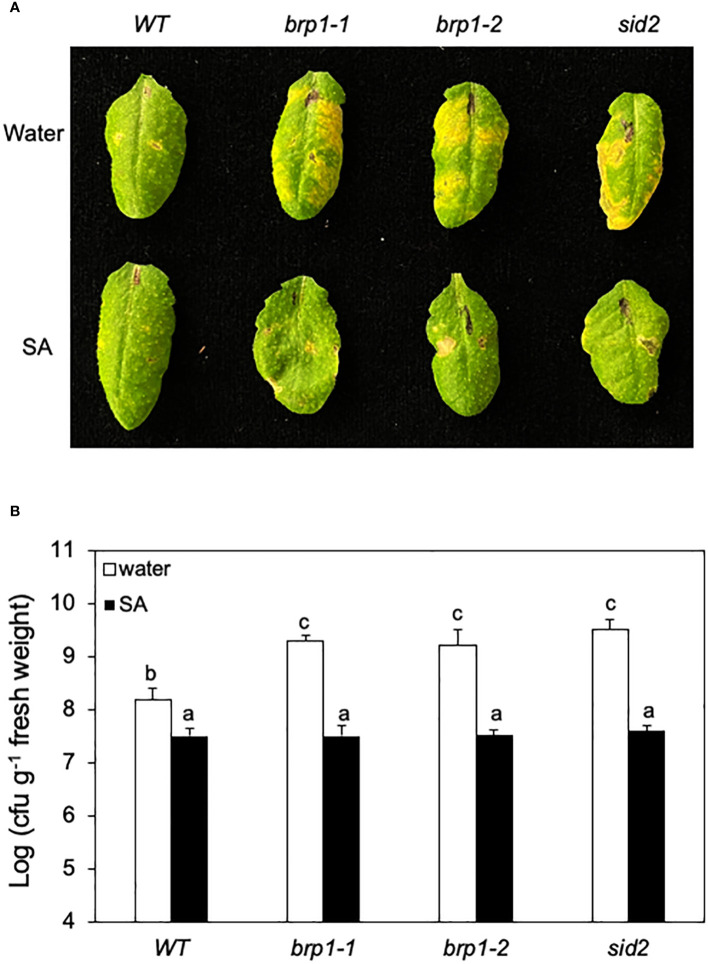
Rescue of *brp1* by SA in disease resistance. **(A)** Six-week-old WT, *brp1* and *sid2* mutants were sprayed with water or SA (1 mM). The plants were infiltrated one day later with a suspension of *Pst*DC3000 (OD_600_ = 0.0002 in 10 mM MgCl_2_). Images are of representative inoculated leaves taken at 4 dpi. **(B)** Effect on bacterial growth. Six-week-old WT and mutants were sprayed with water or SA (1 mM). Pathogen inoculation of WT and mutant plants was performed 1 day later. Samples were taken at 4 dpi to determine the growth of the bacterial pathogen. The means and standard errors were calculated from 6 plants for each treatment. According to Duncan’s multiple range test (P=0.01), means of the values do not differ if they are indicated with the same letter. These experiments were repeated three times with similar results.

The compromised phenotypes of the *brp1* mutants in disease resistance (**Figure 1**) was correlated with reduced expression of SA biosynthetic gene *ICS1/SID2* and SA-regulated *PR* gene expression (**Figure 2**). This correlation suggests that the *brp1* mutants may be defective in SA production. Therefore, we compared the free SA and conjugated SA-glucoside (SAG) levels in WT, *brp1* and *sid2* mutants before and after *Pst*DC3000 infection. At 0 dpi, basal levels of free SA were similar in WT and *brp1* mutants (**Figure 3A**). At 1 dpi, free SA increased by more than 12-fold in WT, but only 4- to 5-fold in the *brp1* mutants (**Figure 3A**). The levels of total SA (free SA and SAG) were already about 4 times lower in the *brp1* mutants than in WT at 0 dpi (**Figure 4B**). At 1 dpi, the levels of total SA in WT were about 6 times higher in WT than in the *brp1* mutants (**Figure 3B**). As previously reported, basal and pathogen-induced free SA and total SA levels in the *sid2* mutant were only about 5 to 10% of those in WT (**Figure 3**).

### Rescue of susceptible phenotype of *brp1* mutants by SA

To determine whether reduced SA accumulation caused disease susceptibility in the *brp1* mutants, we tested whether exogenous SA could restore their disease resistance. We first sprayed WT, *brp1* and *sid2* mutant plants with water or SA, and one day later inoculated the sprayed plants with *Pst*DC3000. After spraying with water, both the *brp1* and *sid2* mutants developed more severe disease symptoms (**Figure 4A**) and supported higher bacterial growth than WT plants did (**Figure 4B**). After SA treatment, however, both the *brp1* and *sid2* mutant had levels of disease symptom development (**Figure 4A**) and bacterial growth similar to those of SA-treated WT plants (**Figure 4B**). These results support that the compromised phenotypes of the *brp1* mutants in disease resistance are caused by reduced SA accumulation after pathogen infection.

### Pathogen-induced nuclear accumulation of BRP1

**Figure 5:**
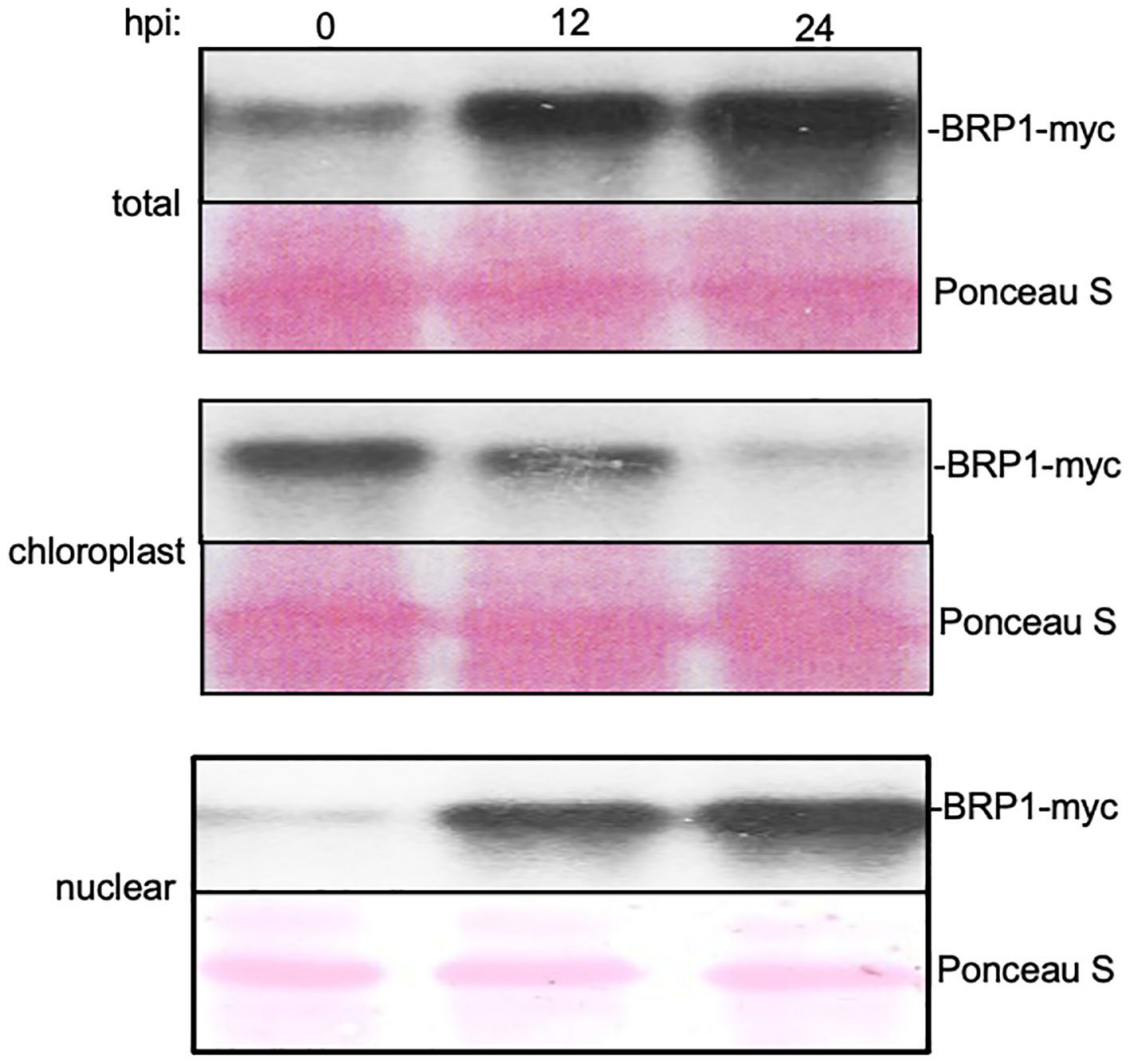
Pathogen-induced nuclear accumulation of BRP1 proteins. Transgenic *brp1-2* mutant plants harboring a genomic *BRP1-myc* gene was inoculated with *Pst*DC3000. Inoculated leaves were sampled at indicated hpi for isolation of chloroplasts and nuclei. BRP1-myc proteins in total (top), chloroplast (middle) and nuclear (bottom) protein extracts were determined by protein blotting using an anti-myc antibody. Ponceau S-stained blots are shown for loading controls. The experiment was repeated twice with similar results.

It has been previously shown that BRP1 is primarily localized to the plastid envelope and its accumulation in the nucleus was detected only after proteasome inhibition (Lagrange et al., 2003). Other published studies reported that co-expression of BRP1-interacting VirE3 transcription activator from *Agrobacterium* promoted nuclear accumulation of BRP1 (Garcia-Rodriguez et al., 2006; Niu et al., 2015). Given the critical role of BRP1 in plant responses to *Pst*DC3000, we analyzed the effect of pathogen infection on its nuclear accumulation. Initially, we generated a BRP1-GFP fusion construct under control of its native promoter and attempted to use confocal microscopy to examine its accumulation and subcellular localization in response to pathogen infection. However, we observed only very low levels of fluorescent signals in the nucleus that were difficult for quantification. Therefore, we generated a 4xmyc-tagged *BRP1* gene under control of its native promoter and investigated the change in both the levels and subcellular localization using subcellular fractionation and protein blotting. The construct was first transformed into the *brp1-2* mutant and was found to fully complement the mutant for resistance to *Pst*DC3000 and, therefore, is fully functional (**Supplementary Figure 2**). Protein blotting using an anti-myc antibody detected very low levels of PBRP-myc in plants at 0 hpi (**Figure 5**). At 12 and 24 hpi, increased levels of BRP1-myc were detected in the inoculated plants (**Figure 5**). RT-qPCR showed that this increase in BRP1-myc proteins was not associated with significant increase in *BRP1* gene transcripts, indicating that pathogen-induced BRP1 protein accumulation involves a post-translational mechanism. We also analyzed the changes of BRP1 protein levels associated with isolated chloroplasts and nuclei from *Pst*DC3000-infected Arabidopsis leaves. Protein blotting of isolated chloroplast and nuclear proteins using antibodies against chloroplast-specific PsbH and nuclear histone H4 proteins revealed little protein cross-contamination in isolated chloroplast and nuclear fractions (**Supplementary Figure 3**). As shown in **Figure 5**, myc-tagged BRP1 in the chloroplast fraction was detected at 0 hpi but significantly reduced at 12 and 24 hpi. By contrast, nuclear BRP1 was barely detectable at 0 hpi but substantially increased at 12 and 24 hpi (**Figure 5**). Thus, pathogen infection increased both the total protein level and nuclear accumulation of BRP1 in plant cells.

### BRP1 as a transcription activator in plant cells

It has been proposed that BRP1 is a general transcription factor for Pol I but not for Pol II primarily based on promoter binding assays of BRP1 from red algae *Cyanidioschyzon merolae* and Arabidopsis (Imamura et al., 2008). However, these reported promoter binding ChIP assays only tested promoters of five light-responsive protein-coding genes as Pol II-dependent promoters, in addition to the promoters of rDNA and 5S rDNA as Pol I- and Pol III-dependent promoters, respectively (Imamura et al., 2008). On the other hand, BRP1 had strong effects on VirE3-depednent expression of plant protein-coding genes (Garcia-Rodriguez et al., 2006; Niu et al., 2015). Furthermore, the normal growth, development and accumulation of rRNAs but compromised phenotypes in disease resistance (**Figure 1**) and defense gene expression (**Figure 2**) in the *brp1* mutants argue against BRP1 as a critical general transcription factor for Pol I.

**Figure 6:**
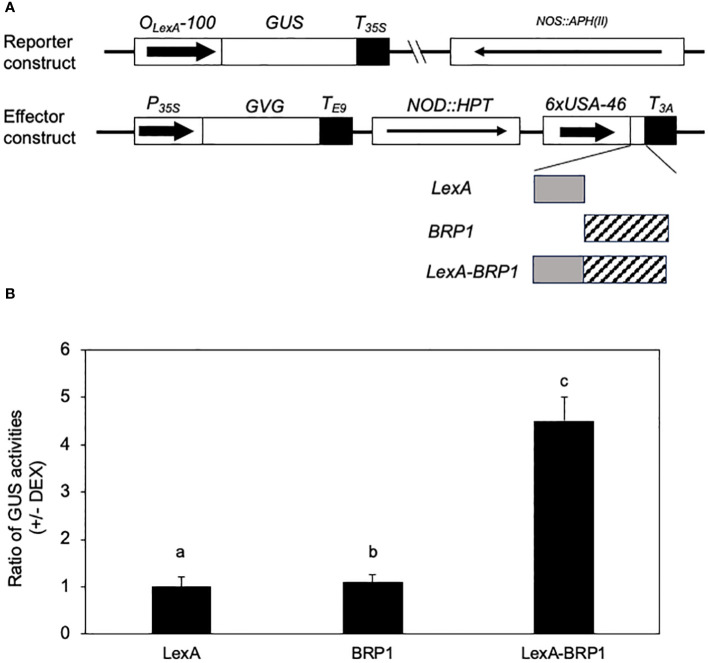
Transcription-activating activity of BRP1 in plant cells. **(A)** Constructs of reporter and effector genes. The *GUS* reporter gene is driven by a synthetic promoter consisting of the -100 minimal *CaMV 35S* promoter and eight copies of the *LexA* operator sequence. The effector genes were clone into pTA7002 behind the steroid-inducible promoter. The three effector genes encode LexADBA-BRP1 fusion protein (LexA-BRP1), LexA DBD (LexA), and BRP1, respectively. **(B)** Effects on the *GUS* reporter gene expression by induced expression of effector genes. The ratios of GUS activities were calculated from the GUS activities in the leaves harvested prior to DEX treatment over those determined in the leaves harvested 18 hours after DEX treatment. The means and errors were calculated from at least 10 positive transformants. According to Duncan’s multiple range test (P=0.01), means of the values do not differ if they are indicated with the same letter. The experiment was repeated twice with similar results.

To determine the role of BRP1 in transcription of protein-coding genes by Pol II in plant cells, we used a previously developed reporter-effector system to analyze the transcriptional regulatory activity of BRP1 through assays of its effects on the protein-coding *GUS* reporter gene in stably transformed plants. In this system, a synthetic promoter consisting of the -100 minimal *CaMV 35S* promoter sequence and eight copies of the *LexA* operator sequence was fused with the *GUS* reporter gene, subcloned into a plant transformation vector, and transformed into Arabidopsis plants (Kim et al., 2006) (**Figure 6A**). These transgenic plants contained low levels of expression of the *GUS* reporter gene due to the minimal *CaMV 35S* promoter, thereby making them possible for assays of transcription activation or repression by determining increase or decrease in GUS activities following coexpression of an effector protein (Kim et al., 2006). To generate the BRP1 effector, we fused its coding sequence with that of the DBD of LexA, subcloned the fusion effector behind the steroid-inducible *Gal4* promoter in pTA7002 (Aoyama and Chua, 1997) and transformed into the transgenic *GUS* reporter lines (**Figure 6A**). Unfused *BRP1* and *LexADBD* genes were also subcloned into pTA7002 and transformed into transgenic *GUS* reporter lines as controls (**Figure 6A**). Transgenic plants containing both the reporter and effector constructs were identified through antibiotic resistance screens and the effects of the effectors on the *GUS* reporter gene expression were determined by assays of the changes of GUS activities following DEX-induced effector gene expression. In the transgenic plants that expressed unfused BRP1 or LexA DBD effector, there was little change in the GUS activities after DEX treatment (**Figure 6B**). These results indicated that induced expression of BRP1 or LexA DBD alone had no significant effect on expression of the *GUS* reporter gene. In the transgenic plants harboring the *LexA DBD-BRP1* effector gene, induction of the fusion effector gene after DEX treatment resulted in approximately 5-fold reduction in GUS activity (**Figure 6B**). These results strongly suggest that BRP1 is a transcriptional activator in plant cells.

### Binding of BRP1 to *ICS1* gene promoter

**Figure 7:**
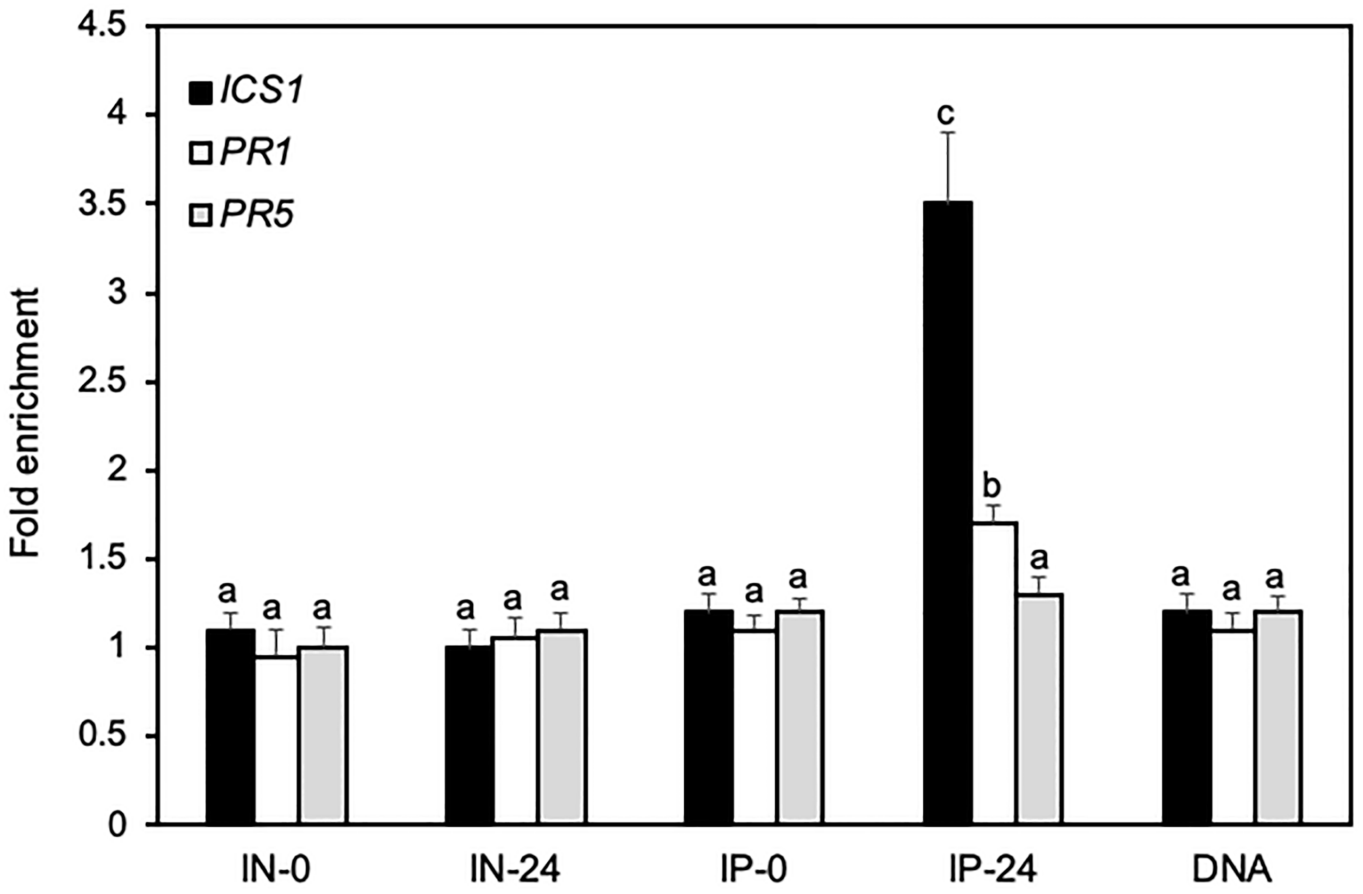
ChIP assays of direct binding of BRP1 to the core promoter elements of defense related genes. Transgenic myc-tagged complementation plants were inoculated with *Pst*DC3000 and inoculated leaves were collected at 0 and 24 hpi and processed for ChIP assays. Input DNA before immunoprecipitation from plant leaves collected at 0 (IN-0) and 24 hpi (IN-24) and coimmunoprecipitated DNA using an anti-myc antibody (IP-0 and IP-24) were analyzed by qPCR using primers specific for the core promoter elements of *ICS*1, *PR1* and *PR5*. The data are expressed as fold enrichment relative to a DNA fragment from RHIP (At4G26410) as a reference gene. Purified genomic DNA (DNA) was also included in the analysis for primer efficiency control. Error bars indicate SE (n = 3). According to Duncan’s multiple range test (P=0.01), means of the values do not differ if they are indicated with the same letter. The experiment was repeated twice with similar results.

As a TFIIB-related protein, BRP1 does not necessarily bind DNA directly but could recognize the core promoter elements of its target genes such as TATA boxes through associated TBPs as a general transcription factor of Pol II. Since BRP1 positively regulates pathogen-induced expression of *ICS1*, *PR1* and *PR5*, we performed ChIP using the myc-tagged BRP1 complementation lines in combination with qPCR to determine direct BRP1 binding to the core promoter regions of these defense-related genes. By using primer sets representing the core promoter regions (~100 nucleotides upstream of the putative transcript start sites) of the defense-related genes (**Supplementary Table 3**), we detected significant pathogen-dependent binding of BRP1 to the promoter of *ICS1* (**Figure 7**). We also observed weak but significant binding of BRP1 to the core promoter region of *PR1* (**Figure 7**). On the other hand, no significant binding of BRP1 to the core promoter region of *PR5* was detected (**Figure 7**). These results indicated that BRP1 directly regulated expression of specific defense-related genes during plant defense responses.

## Discussion

There are 15 genes encoding TFIIB-like factors in the Arabidopsis genome (Knutson, 2013; Ning et al., 2021). Two of them (*BRF4CTD* and *MEE12CTD*) encode proteins that lack the conserved N-terminal zinc finger and the two cyclin fold repeats of TFIIB and are unlikely to act as TFIIB-like factors (Ning et al., 2021). Of the remaining 13 Arabidopsis TFIIB-like factors, at least 10 have been characterized through molecular genetic approaches and found to play important roles in gametogenesis, pollen tube growth guidance, embryogenesis, and endosperm development (Ning et al., 2021). Thus, the expansion and functional diversification of the TFIIB-related proteins in plants may contribute to the evolution of novel functions associated with plant-specific sexual reproductive processes. As sessile organisms, plants are also constantly exposed to a wide spectrum of biotic and abiotic stress conditions and have also evolved many unique stress- and defense-response mechanisms. Previously, Arabidopsis BRP1 has been implicated in the interaction between plants and *Agrobacterium* (Garcia-Rodriguez et al., 2006; Niu et al., 2015; Li et al., 2021a). In the present study, we have provided direct genetic evidence that the plant-specific TFIIB-related protein plays a critical role in plant defense responses.

Using T-DNA insertion and CRISPR/cas9 genome editing, we have generated two independent knockout mutants for Arabidopsis BRP1 (**Figure 1A**). Unlike knockout mutants for other characterized Arabidopsis TFIIB-related proteins, which are either lethal or severely compromised in important reproductive processes, the *brp1* mutants displayed no significant phenotypes in growth and development. However, the *brp1* mutants are highly susceptible to the bacterial pathogen *P. syringae* based on both enhanced symptoms and increased pathogen growth (**Figure 1**). Further analysis indicated that the hyper-susceptibility of the *brp1* mutants to the bacterial pathogen was caused by compromised SA accumulation in association with reduced expression of SA biosynthetic gene *SID2/ICS1* (**Figures 2**, **3**). ChIP assays further indicated that BRP1 directly regulates pathogen-induced expression of *SID2ICS1* (**Figure 7**). SA is an important defense signal with a critical role in both basal disease resistance and systemic acquired resistance mostly through transcriptional regulation of transcription program of plant defense genes (Seyfferth and Tsuda, 2014; Yan and Dong, 2014). Indeed, compromised disease resistance of the *brp1* mutants was also correlated with defects in SA-regulated *PR1* and *PR5* gene expression (**Figure 2**). Thus, the plant-specific TFIIB-related protein has an important and specific role in plant immunity by promoting SA-mediated defense responses.

Previously, it has been claimed that plant-specific BRP1 functions as a general transcription factor for Pol I, but not for Pol II or III (Imamura et al., 2008). This role of plant-specific BRP1 was primarily based on the binding of BRP1 to the Pol I-dependent rDNA promoters both *in vitro* and *in vivo* (Imamura et al., 2008). The reported study argued against a role of BRP1 in Pol II-dependent transcription because it failed to detect its binding to the promoters of five light-responsive protein-coding genes as Pol II-dependent promoters (Imamura et al., 2008). If BRP1 functions as a critical general transcription factor for Pol I responsible for transcription of rDNA, which accounts for over 50% of total cellular RNA, we expect that the Arabidopsis *brp1* knockout mutants would display severe or even lethal phenotypes with greatly reduced levels of rRNAs. The lack of significant phenotypes in growth and development as well as the normal accumulation of rRNAs in the Arabidopsis *brp1* knockout mutants strongly argues against a critical role of the plant-specific TFIIB-related protein in Pol I-dependent transcription. It is possible that BRP1 is involved in transcription by Pol I and the lack of effects from the loss of BRP1 on rRNA accumulation is resulted from the presence of additional TFIIB-related proteins as general transcription factor of Pol I.

On the other hand, Arabidopsis *brp1* mutants were compromised in plant immunity due to defects in expression of genes involved in SA biosynthesis and other defense-related processes (**Figures 1**, **2**). Furthermore, BRP1 is associated with the core promoter elements of *ICS1* and, to a less extent, *PR1* (**Figure 7**). Previously, it has been shown that plant BRP1 interacts with the virE3 transcription factor from *Agrobacterium* and promotes transcription of a large number of virE3-activated host protein-coding genes to promote *Agrobacterium*-mediated transformation (Garcia-Rodriguez et al., 2006; Niu et al., 2015; Li et al., 2021a). In addition, we have shown that BRP1 directly activated transcription of the protein-coding *GUS* reporter gene in plant cells (**Figure 6**). Therefore, plant-specific BRP1 functions as a critical transcription activator of plant genes involved in plant-microbe interactions and is targeted by plant pathogens such as *Agrobacterium* during the infection process to modulate plant host gene expression.

The critical role of BRP1 in plant-microbe interaction is consistent with the highly regulated nature of its subcellular localization. As previously reported (Lagrange et al., 2003), Arabidopsis BRP1 is primarily associated with chloroplasts in the absence of pathogen infection (**Figure 5**). Increased nuclear accumulation of BRP1 has been observed in plant cells after inhibition of proteasome or in COP9 mutants (Lagrange et al., 2003). Under normal growth condition, its nuclear accumulation is very limited probably through inhibition of nuclear translocation and the degradation of nuclear BRP1. The limited nuclear accumulation of BRP1 may be necessary to prevent unnecessary activation of defense-related genes. After infection by *Pst*DC3000 infection, however, there was increased accumulation of BRP1 in the nucleus, concomitant with its decreased association in chloroplasts (**Figure 5**). Increased nuclear accumulation of BRP1 has also been observed in virE3-coexpressed cells (Garcia-Rodriguez et al., 2006). Bioinformatics analysis finds no nuclear localization signal in the protein, suggesting that the nuclear localization of BRP1 may be mediated by a piggyback mechanism through interaction with a nuclear protein such as *Agrobacterium* virE3, effector proteins from *P. syringae* and other plant host proteins. Further investigation of the mechanisms by which the subcellular localization of BRP1 is dynamically regulated under both normal and stress conditions could provide important new insights into the complex network of molecular events that balance plant growth with plant stress/defense responses.

Establishment of a TFIIB-related general transcription factor such as BRP1 in specific biological processes is highly significant as the findings challenge the paradigm of general transcription factors as universal regulators of class-specific gene expression. Structural and evolutionary analysis has shown that bacterial σ factors, archaeal transcription factor B (TFB) and eukaryotic TFIIB are homologs (Burton and Burton, 2014). Bacteria often contain a primary σ factor and many alternative σ factors for regulation of discrete sets of genes (Burton and Burton, 2014; Feklistov et al., 2014). Archaea also have multiple TFB factors that potentially mediate environmental responses, which may explain their extraordinary niche adaptation capability. In *Halobacterium salinarum*, a halophilic (salt-loving) member of the Archaea that grows in concentrations of NaCl near or at saturation, there are at least seven TFBs that direct environment-specific gene expression programs (Turkarslan et al., 2011). In eukaryotes, the functions of general transcription factors have been analyzed almost exclusively in the context of basal transcription and their possible roles in the regulation of physiology may have been under-appreciated. In yeast, ethanol production could be enhanced through the mutagenesis of TFIIB, suggesting that altering the function of a general transcription factor can have significant phenotypic consequences (D'Alessio et al., 2009). Furthermore, several studies have discovered regulatory roles of general transcription factors in cell-specific differentiation and development in eukaryotes (Lagrange et al., 2003; Juven-Gershon and Kadonaga, 2010). The large number of TFIIB-related proteins and their distinct roles in a broad spectrum of plant sexual reproduction and disease resistance present a new paradigm for the transcription and transcriptional regulation of genes, which can provide novel insights into the transcriptional programs that govern plant growth, development and responses to both biotic and abiotic stresses.

## Accession numbers

The identifiers for the Arabidopsis genes described in this article are as follows: *BRP1* (AT4G36650), *ICS1/SID2* (AT1G74710), *PR1* (AT2G14610), *PR5* (AT1G75040), *Actin2* (AT3G18780), PsbH (ATCG00710), Histine H4 (AT2G28740 and RHIP (AT4G26410).

## Data availability statement

The raw data supporting the conclusions of this article will be made available by the authors, without undue reservation.

## Author contributions

BX: Writing – review & editing, Data curation, Investigation. BF: Investigation, Writing – review & editing. ZC: Conceptualization, Funding acquisition, Writing – original draft, Writing – review & editing.

## Supplementary material

Supplementary Figure 1 Characterization of Arabidopsis *brp1* mutants. **(A)** Expression of *BRP1* in WT and *brp1-1* mutant. Total RNA was isolated from leaf samples collected from six-week-old plants. Transcript levels of *BRP1* were determined using RT-qPCR. Error bars indicate SE (n = 3). A Student’s *t*-test was used for statistical analysis of the *BRP1* transcript levels in WT versus in *brp1-1* mutant (***indicates p-value < 0.001). **(B)** Predicted effect of the *brp1-2* mutation on the translated product of *BRP1*. A single A base insertion between nucleotides 232 and 233 of the *BRP1* coding sequence would cause a reading frame shift after amino acid residue 77 and introduce a premature termination codon after addition of 29 wrong amino acid residues (in red).

Supplementary Figure 2 Complementation of *brp1-2* by a myc-tagged *BRP1* gene. **(A)** Six-week-old WT, *brp1-2* mutant and two independent lines (L1 and L2) of *brp1-2* mutant expressing a myc-tagged *BRP1* gene under its native promoter were infiltrated with a suspension of *Pst*DC3000 (OD_600_ = 0.0002 in 10 mM MgCl_2_). Images are of representative inoculated leaves taken at 4 dpi. **(B)** Effect on bacterial growth. Six-week-old WT, *brp1-2* mutant and two independent lines (L1 and L2) of *brp1-2* mutant expressing a myc-tagged *BRP1* gene under its native promoter were infiltrated with a suspension of *Pst*DC3000 (OD_600_ = 0.0002 in 10 mM MgCl_2_). Samples were taken at 4 dpi to determine the growth of the bacterial pathogen. The means and standard errors were calculated from 6 plants for each treatment. According to Duncan’s multiple range test (P=0.01), means of the values do not differ if they are indicated with the same letter. These experiments were repeated twice times with similar results.

Supplementary Figure 3 Protein blotting for detection of potential cross-contamination of isolated chloroplast and nuclear fractions. Transgenic *brp1-2* mutant plants harboring a genomic *BRP1-myc* gene was inoculated with *Pst*DC3000. Inoculated leaves were sampled at indicated hpi for isolation of chloroplasts and nuclei. The same amount of proteins from each chloroplast and nuclear fraction was fractionated by electrophoresis and analyzed by protein blotting using an anti-PsbH or anti-histone H4 (H4) antibody. The experiment was repeated twice with similar results.
