# Supplementary figures and images for: A critical role of a plant-specific TFIIB-related protein, BRP1, in salicylic acid-mediated immune response
Source: Front Plant Sci. 2024 Jul 30;15:1427916. doi: 10.3389/fpls.2024.1427916 (PMC11319285; doi:10.3389/fpls.2024.1427916)

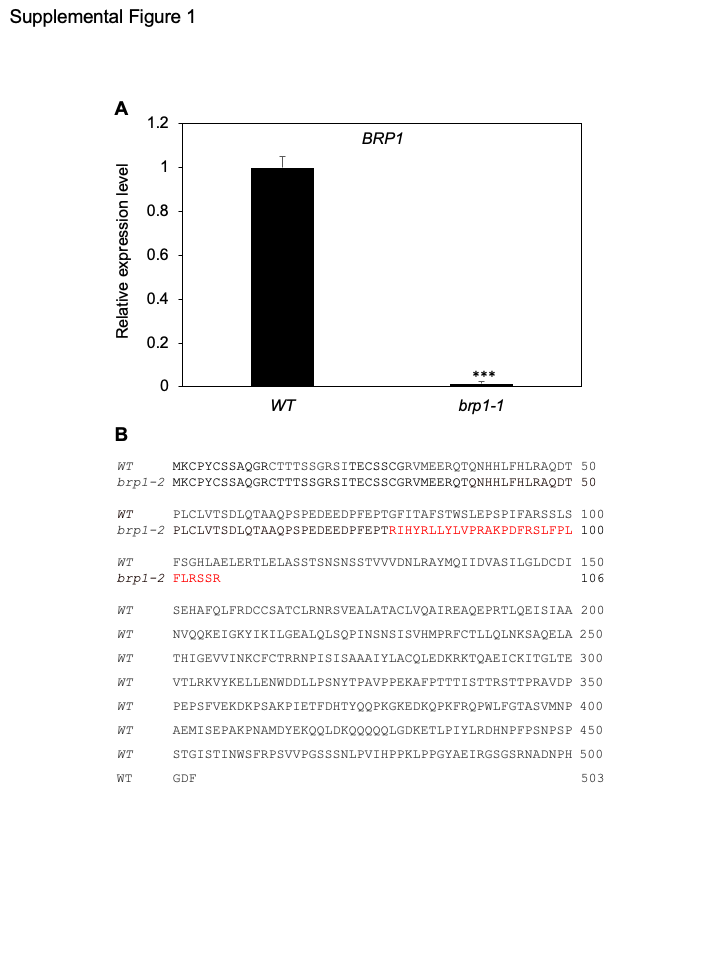

Supplement: Supplementary Figure 1 — Characterization of Arabidopsis brp1 mutants. (A) Expression of BRP1 in WT and brp1-1 mutant. Total RNA was isolated from leaf samples collected from six-week-old plants. Transcript levels of BRP1 were determined using RT-qPCR. Error bars indicate SE (n = 3). A Student’s t-test was used for statistical analysis of the BRP1 transcript levels in WT versus in brp1-1 mutant (***indicates p-value < 0.001). (B) Predicted effect of the brp1-2 mutation on the translated product of BRP1. A single A base insertion between nucleotides 232 and 233 of the BRP1 coding sequence would cause a reading frame shift after amino acid residue 77 and introduce a premature termination codon after addition of 29 wrong amino acid residues (in red). [file Image_1.tiff]

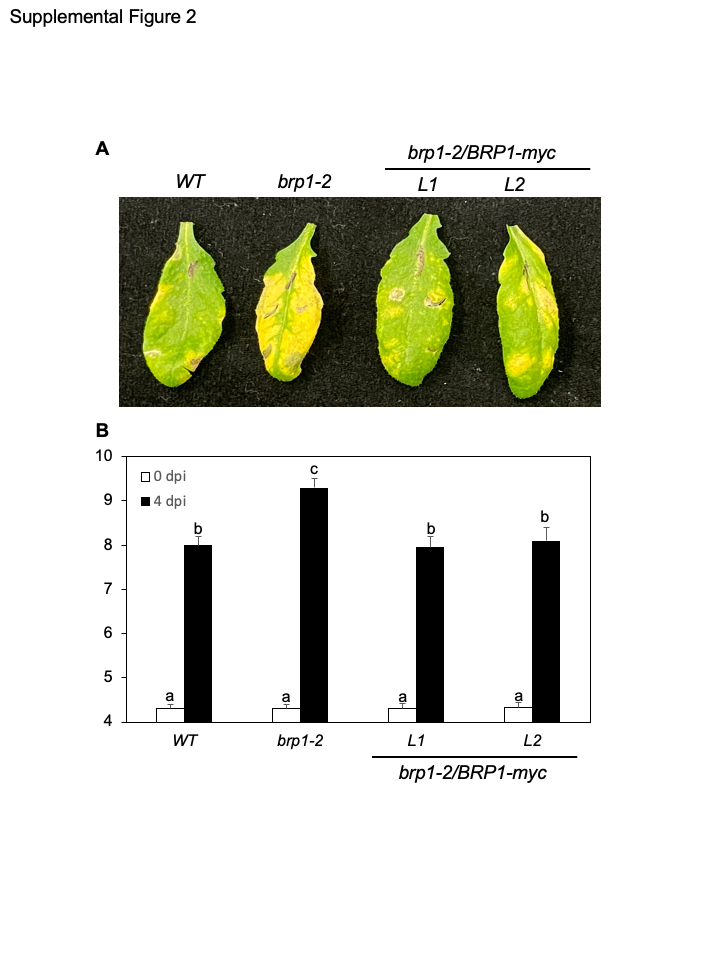

Supplement: Supplementary Figure 2 — Complementation of brp1-2 by a myc-tagged BRP1 gene. (A) Six-week-old WT, brp1-2 mutant and two independent lines (L1 and L2) of brp1-2 mutant expressing a myc-tagged BRP1 gene under its native promoter were infiltrated with a suspension of PstDC3000 (OD600 = 0.0002 in 10 mM MgCl2). Images are of representative inoculated leaves taken at 4 dpi. (B) Effect on bacterial growth. Six-week-old WT, brp1-2 mutant and two independent lines (L1 and L2) of brp1-2 mutant expressing a myc-tagged BRP1 gene under its native promoter were infiltrated with a suspension of PstDC3000 (OD600 = 0.0002 in 10 mM MgCl2). Samples were taken at 4 dpi to determine the growth of the bacterial pathogen. The means and standard errors were calculated from 6 plants for each treatment. According to Duncan’s multiple range test (P=0.01), means of the values do not differ if they are indicated with the same letter. These experiments were repeated twice times with similar results. [file Image_2.tiff]

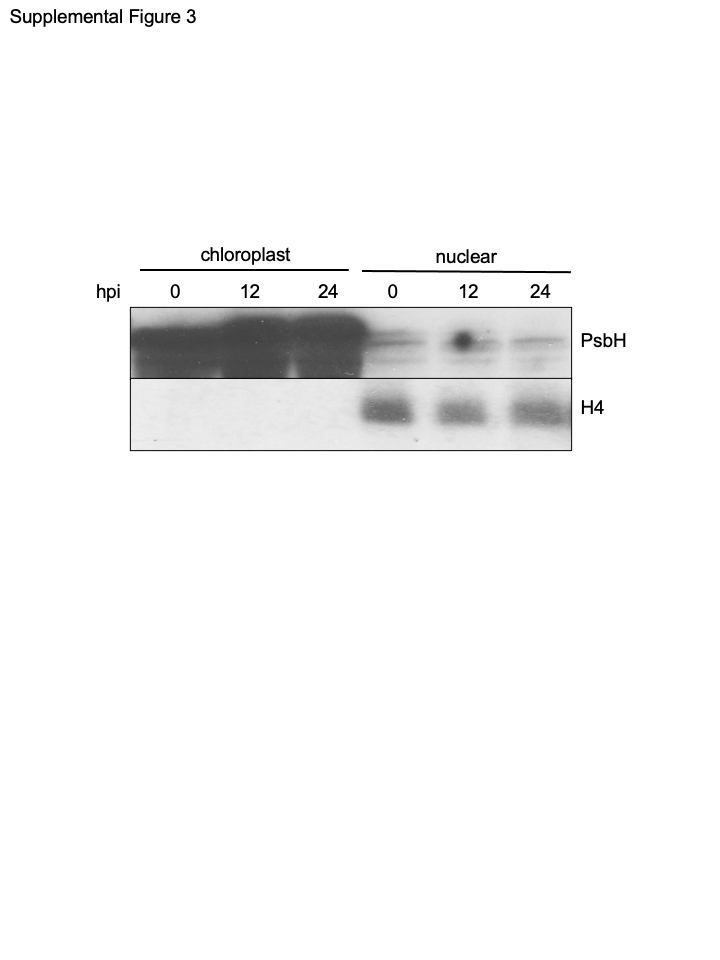

Supplement: Supplementary Figure 3 — Protein blotting for detection of potential cross-contamination of isolated chloroplast and nuclear fractions. Transgenic brp1-2 mutant plants harboring a genomic BRP1-myc gene was inoculated with PstDC3000. Inoculated leaves were sampled at indicated hpi for isolation of chloroplasts and nuclei. The same amount of proteins from each chloroplast and nuclear fraction was fractionated by electrophoresis and analyzed by protein blotting using an anti-PsbH or anti-histone H4 (H4) antibody. The experiment was repeated twice with similar results. [file Image_3.tiff]
