# Supplementary material for: A critical role of a plant-specific TFIIB-related protein, BRP1, in salicylic acid-mediated immune response
Source: Front Plant Sci. 2024 Jul 30;15:1427916. doi: 10.3389/fpls.2024.1427916 (PMC11319285; doi:10.3389/fpls.2024.1427916)
Supplement: Supplementary file 4 [file Table_1.docx]

**Supplemental Table 1.** Primers for genotyping of Arabidopsis *brp1* and *sid2* mutants.

| mutant | Primer sequence |
| --- | --- |
| *brp1-1* | 5’-cccgcttccaatgactttt-3’  5’-tgctgctgcttatcgagttg-3’ |
| *brp1-2* | 5’-cacaacttctttcaactcaaaacg-3’  5’-gaagtgtccgtactgttcatcg-3’ |
| *sid2* | 5’-caatccgatttgctgctgta-3’  5’-gcatggccacactaactaaaa-3’ |

**Supplemental Table 2**. Primers for RT-qPCR analysis

| Gene | Primer sequence |
| --- | --- |
| *BRP1* | 5’-caagcgaaagacacaagcag-3’  5’-tgggaaatgctttctctggt-3’ |
| *ICS1/SID2* | 5’-tgtctgcagtgaagctttgg-3’  5’-gaactcgaggtcgtctttcg-3’ |
| *PR1* | 5’-ggccttacggggaaaactta-3’  5’-caaactccattgcacgtgtt-3’ |
| *PR5* | 5’-cgtacaggctgcaactttga-3’  5’-tgaattcagccagagtgacg-3’ |
| *Actin2* | 5’-gccatccaagctgttctctc-3’  5’-cagtaaggtcacgtccagca-3’ |

**Supplemental Table 3**. Primers for ChIP-qPCR analysis

| Gene | Primer sequence |
| --- | --- |
| *ICS1/SID2* | 5’-tctgagaaattttgtgtgaaaatgaa-3’  5’-tttggtttgggtttagagttgaga-3’ |
| *PR1* | 5’-cgaaaacaacttctattcatttgga -3’  5’-ttgttaactatagatctcacgtttttg-3’ |
| *PR5* | 5’-ctgcaaagaaaattcagagaacca-3’  5’-atgttggttcgaggggtagc-3’ |
| *RHIP* | 5’-agcgatcggagtggttgaat-3’  5’-tgcggtatccttcgattcct-3’ |
